# Case report: Susac syndrome—two ends of the spectrum, single center case reports and review of the literature

**DOI:** 10.3389/fneur.2024.1339438

**Published:** 2024-02-16

**Authors:** Martina Cviková, Jakub Štefela, Vít Všianský, Michal Dufek, Irena Doležalová, Jan Vinklárek, Roman Herzig, Markéta Zemanová, Vladimír Červeňák, Jaroslav Brichta, Veronika Bárková, David Kouřil, Petr Aulický, Pavel Filip, Viktor Weiss

**Affiliations:** ^1^Department of Neurology, St. Anne’s University Hospital in Brno and Faculty of Medicine at Masaryk University, Brno, Czechia; ^2^Department of Neurology, Comprehensive Stroke Center, Charles University Faculty of Medicine and University Hospital, Hradec Králové, Czechia; ^3^Department of Ophthalmology and Optometry, St. Anne’s University Hospital in Brno and Faculty of Medicine at Masaryk University, Brno, Czechia; ^4^Department of Medical Imaging, St. Anne’s University Hospital and Faculty of Medicine, Masaryk University, Brno, Czechia; ^5^Department of Clinical Pharmacology, St. Anne's University Hospital, Brno, Czechia; ^6^Department of Neurology, Blansko Hospital, Blansko, Czechia; ^7^Department of Anesthesiology, Hospital of the Brothers of Charity Brno, Brno, Czechia; ^8^Department of Neurology, Charles University, First Faculty of Medicine and General University Hospital, Prague, Czechia; ^9^Center for Magnetic Resonance Research (CMRR), University of Minnesota, Minneapolis, MN, United States; ^10^Department of Neurology, Charles University Faculty of Medicine, Hradec Králové, Czechia

**Keywords:** Susac syndrome, vasculitis, branch retinal arterial occlusion, stroke, neuroimmunology, black blood imaging, hearing loss

## Abstract

Susac syndrome is a rare and enigmatic complex neurological disorder primarily affecting small blood vessels in the brain, retina, and inner ear. Diagnosing Susac syndrome may be extremely challenging not only due to its rarity, but also due to the variability of its clinical presentation. This paper describes two vastly different cases—one with mild symptoms and good response to therapy, the other with severe, complicated course, relapses and long-term sequelae despite multiple therapeutic interventions. Building upon the available guidelines, we highlight the utility of black blood MRI in this disease and provide a comprehensive review of available clinical experience in clinical presentation, diagnosis and therapy of this disease. Despite its rarity, the awareness of Susac syndrome may be of uttermost importance since it ultimately is a treatable condition. If diagnosed in a timely manner, early intervention can substantially improve the outcomes of our patients.

## Introduction

Susac syndrome (SuS) is an autoimmune endotheliopathy, which affects precapillary segments of arteries in the brain, cochlea, and retina. Resulting in a clinical triad of encephalopathy, sensorineural hearing loss, and visual loss (1–3). SuS is a rare and often underdiagnosed condition with about 450 cases reported worldwide. It affects young people, especially women (4). Clinical presentation is variable (2, 5–7) and not all patients exhibit the whole triad at the onset of symptoms (2, 4–9). In addition to clinical variability, the disease course and aggression vary widely (1). In mild cases, peak disease severity lasts only for several months, and the dysfunction is reversible. On the other hand, prolonged course over several years with devastating long-term or even fatal consequences has been reported as well (1, 8, 10, 11).

Due to variable presentation, delayed diagnosis, and variable course of the disease, the management is very challenging as well (1–3, 8). Low disease prevalence precludes controlled randomized clinical trials and treatment guidelines are based on the approaches generally utilized in other autoimmune diseases (1, 9).

Only one case of SuS has been reported from the Czech Republic so far. This paper describes further two patients admitted to our Neurology department within 6 months—the first one presenting with mild symptoms, the second one at the other one end of the clinical spectrum including very rare cardiac involvement (6), where all of the available treatment options (corticosteroids, cyclophosphamide, intravenous immunoglobulins, plasmapheresis, and rituximab) were used to achieve remission. And lastly, we provide a complex updated review of Susac syndrome to create a sound basis for the management of further patients.

## Case reports

### Patient 1

**Figure 1:**
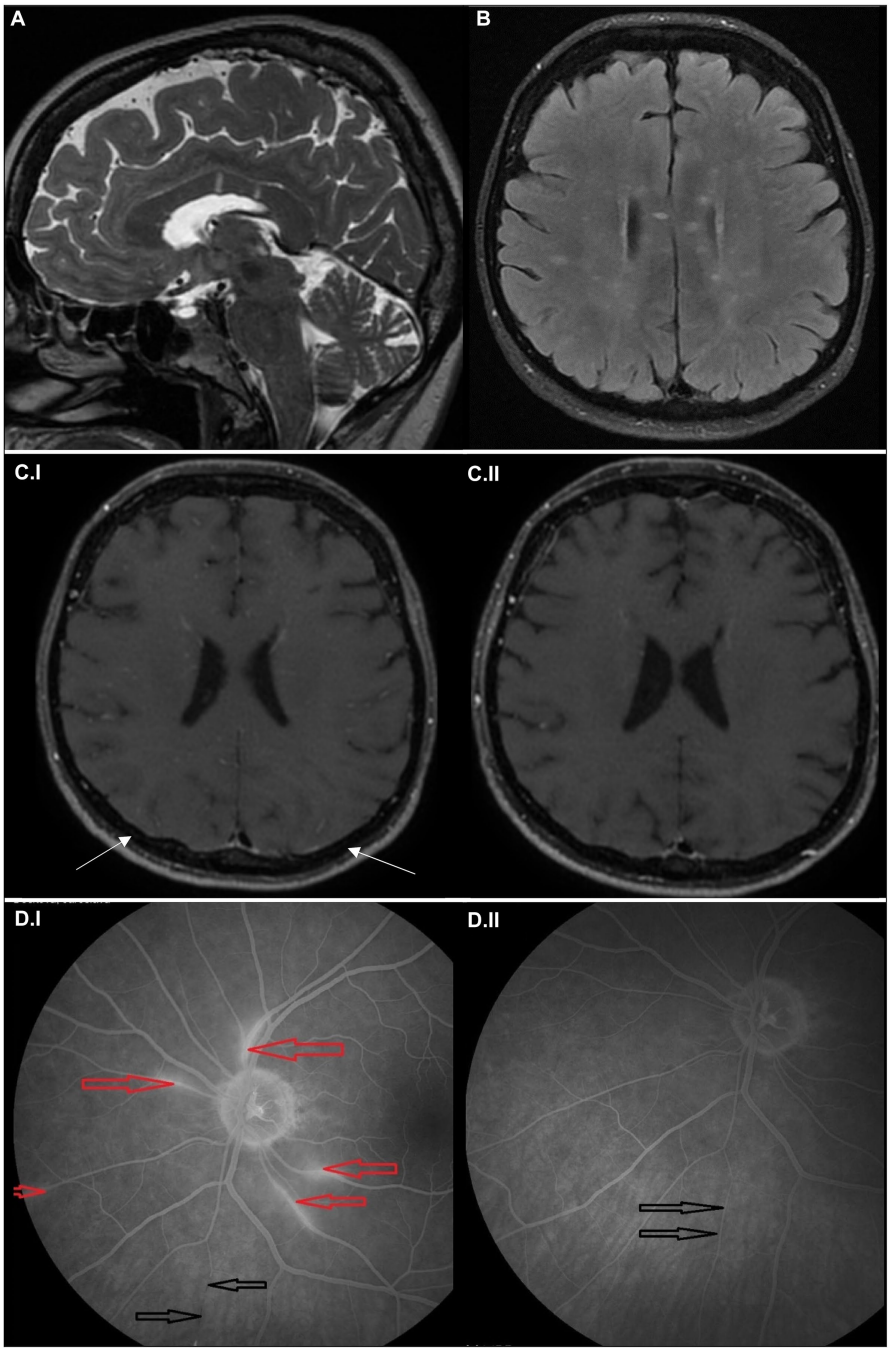
Imaging findings in the case report 1. Sagittal T2-weighted **(A)** and axial FLAIR MRI scans **(B)** at the time of symptom onset showing multiple hyperintense small lesions in the white matter, predominantly affecting corpus callosum in the form of “spokes.” Axial black blood MRI scans at the time of symptom onset **(C.I)**, showing microangiopathy as multiple small, hypersignal nodules in small vessel walls combined with discreet leptomemingeal enhancement (marked with arrows); and after 6 months **(C.II)**, showing regression of the pathology. Fluorescein angiogram (left eye) at the time of symptom onset **(D.I)**, where the areas of hyperfluorescence mark leakage from inflamed retinal arterioles (red arrows) and the area of fluorescence decrease corresponds to the lower nasal arteriolar occlusion (precapillary plaque) with segmentation (black arrows). Fluorescein angiogram (left eye) after 8 months **(D.II)** shows the absence of leakage and reperfusion of the previously occluded retinal arterioles (black arrows).

A 61-year-old woman was admitted to the hospital for acute onset of rotatory vertigo, vomiting, and confusion lasting for several hours. At the time of admission, neurological examination revealed only partial amnesia without any focal neurological deficit. Initial brain computer tomography (CT), CT angiography, and brain CT perfusion scans were normal. Magnetic resonance (MRI) scans of the head showed several lesions (Figures 1A,B) in the corpus callosum and in the left precuneus with diffusion restriction; black blood MRI (Figure 1C.I) detected inflammatory microangiopathy, with gadolinium enhancement of the small vessel walls, dot-like infarctions, and focal leptomeningeal enhancement. Lumbar puncture revealed mild mononuclear pleocytosis (eight lymphocytes/μL, no neutrophiles, and no red blood cells) and mild protein elevation (1,623 mg/L), no oligoclonal bands were found. Extensive screening failed to find any infectious etiology. Audiometry showed sensorineural hearing loss maximal at 125 and 500 Hz on the right ear. Ophthalmologic exam including fundoscopy and visual field test appeared completely normal, no vascular changes of the retina were found. Due to a suspicion of SuS, fundus fluorescein angiography (FAG) was performed, detecting regional hyperfluorescence and branch retinal artery occlusion with segmentation (Figure 1D). Since the findings were consistent with suspected SuS, the patient was treated with a pulse of methylprednisolone (total dose of 5 g) followed by high-dose oral prednisone (1 mg/kg) with a slow taper. Over time, cognitive impairment normalized while hearing impairment on the right ear persisted. Due to the stable course of the disease patient was started on azathioprine (100 mg per day, 1.3 mg/kg). Brain MRI after 6 months did not show any new lesions with diffusion restriction. Black blood MRI (Figure 1C.II) revealed regression of leptomeningeal and vessel wall enhancement.

### Patient 2

**Figure 2:**
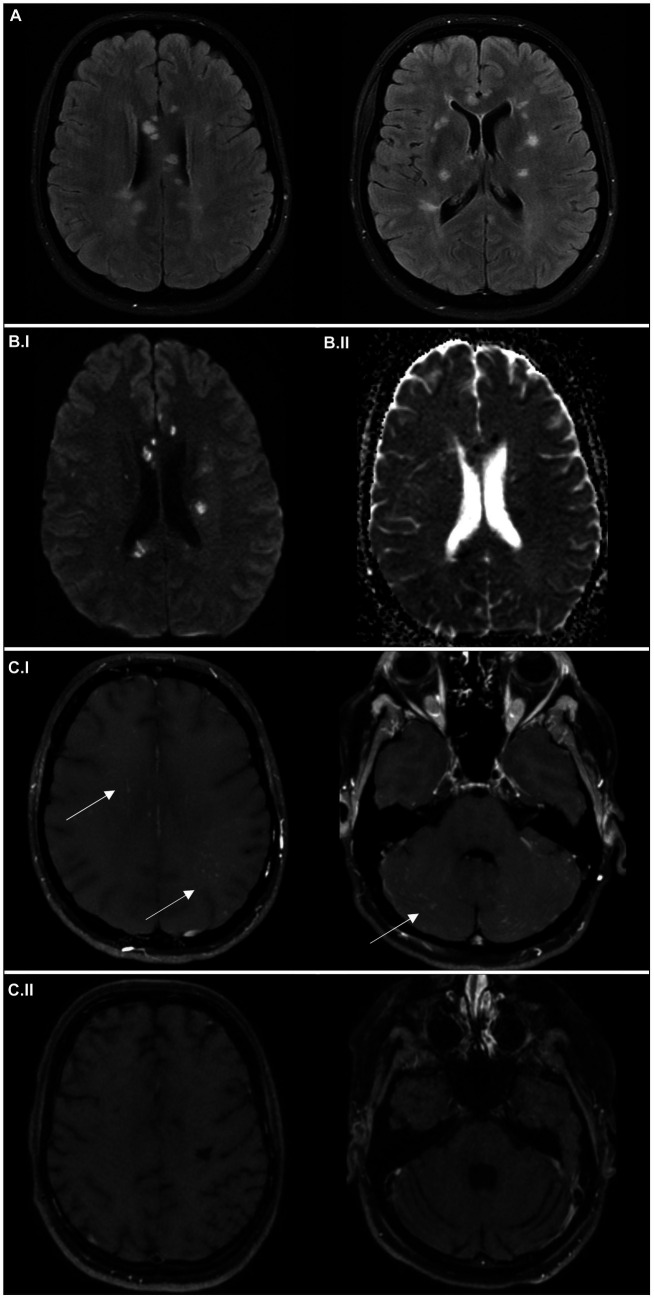
Imaging findings in the case report 2. Axial FLAIR MRI scans **(A)** at the time of symptom onset showing multiple hyperintense “snowball” lesions in the white matter. Axial average diffusion-weighted scans **(B.I)** at the time of symptom onset and apparent diffusion coefficient maps **(B.II)**, with small areas of restriction of diffusion (hypersignal in **B.I** and hyposignal in **B.II**), corresponding to small infarctions. Black blood MRI scans at the time of symptom onset **(C.I)**, detecting microangiopathy (multiple small hypersignal nodules of small vessel walls) in both supra- and infratentorial region; and after 6 months **(C.II)**, with regression of the initial pathology, but development of substantial atrophy.

A 32-year-old woman presented with recurrent weakness and paresthesia of left-side limbs. Initially, she was admitted to a stroke unit of a local hospital and later transferred to our neurology department due to the deterioration of her clinical condition. Initial neurological examination indicated severe encephalopathy, with multiple domain cognitive impairment, left-sided upper motor neuron syndrome, and tactile neglect syndrome. ECG monitoring showed persistent asymptomatic bradycardia. Echocardiogram and cardiologic examination did not reveal any specific cause. Brain MRI detected multiple areas of restriction of diffusion predominantly in the corpus callosum, in bilateral subcortical white matter and in the cerebellum (Figure 2). Black blood MRI showed gadolinium enhancement of small vessel walls (Figure 2C.I), similarly to the first patient. There was also focal leptomeningeal gadolinium-enhancement in the posterior fossa. In the cerebrospinal fluid (CSF), mild mononuclear pleocytosis (14 lymphocytes/μL, no neutrophiles, and six red blood cells/μL) and elevated protein concentration (1,700 mg/L) were found; no oligoclonal bands were present. Infectious and systemic autoimmune etiology was excluded. Audiometry confirmed subclinical low frequency sensorineural hearing loss in the left ear. Fundoscopy detected intraretinal hemorrhage and peripheral artery occlusion in the left eye. Unfortunately, FAG was not performed at this stage before the initiation of the therapy.

In the first line, the patient was treated with two pulses of methylprednisolone (5 g for the first pulse and 7 g for the second pulse after a clinical and radiological relapse) followed by a taper (80 mg of prednisone per day). At the same time, plasmapheresis was performed as an alternative to intravenous immunoglobulins (IVIG). However, the patient developed coagulopathy and the treatment had to be prematurely terminated after 4 cycles. After clinical and radiological stabilization, cyclophosphamide was administered intravenously (12 mg/kg) in a single pulse, after that, cyclophosphamide was continued orally at 100 mg/day. The treatment led to clinical improvement, subsequent brain MRI showed regression of the lesions and no gadolinium-enhancement of vessel walls or leptomeninges. Furthermore, bradycardia resolved. FAG performed at this stage showed multiple branch retinal artery occlusions and some areas of hyperfluorescence (Figure 4A).

**Figure 3:**
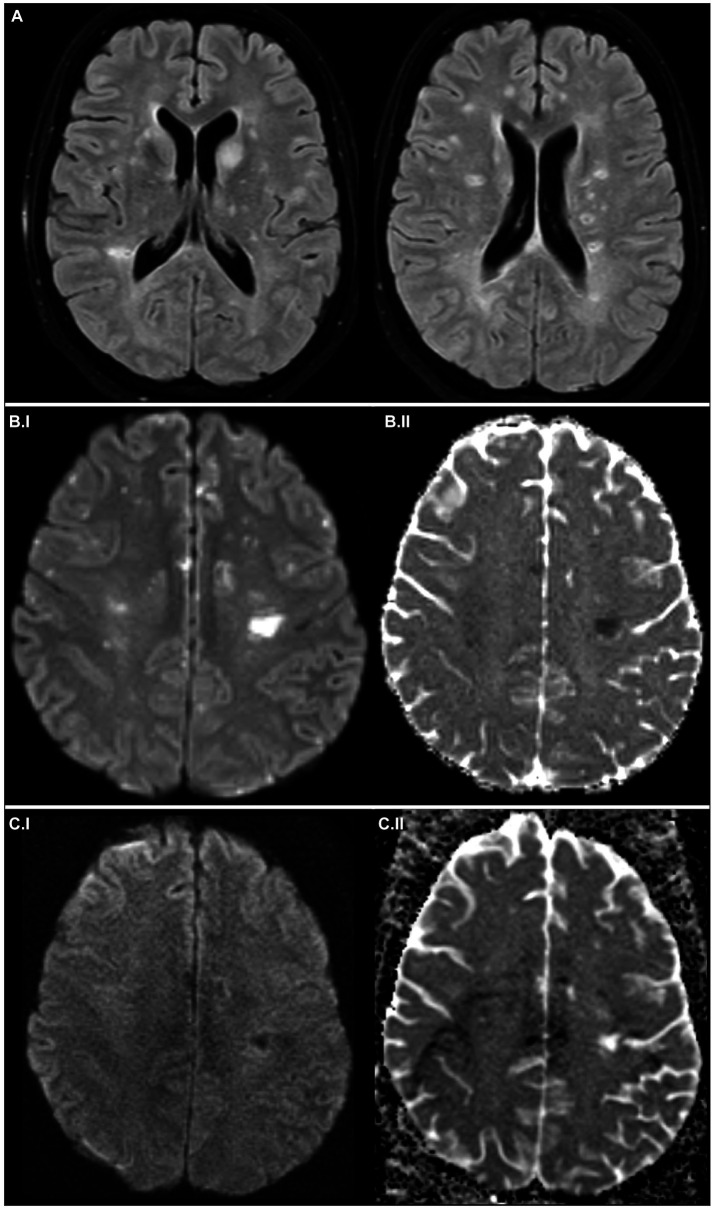
Imaging findings in the case report 2. Axial FLAIR MRI scans **(A)** about 1.5 months after the symptom onset, showing cystoid degeneration of previous hypersignal areas and several new lesions. Axial average diffusion-weighted scans **(B.I)** and apparent diffusion coefficient maps **(B.II)** about 1.5 months after the symptom onset, with several new lesions causing diffusion restriction, and 6 months after the symptom onset **(C.I,C.II)**, where no new lesions are apparent.

**Figure 4:**
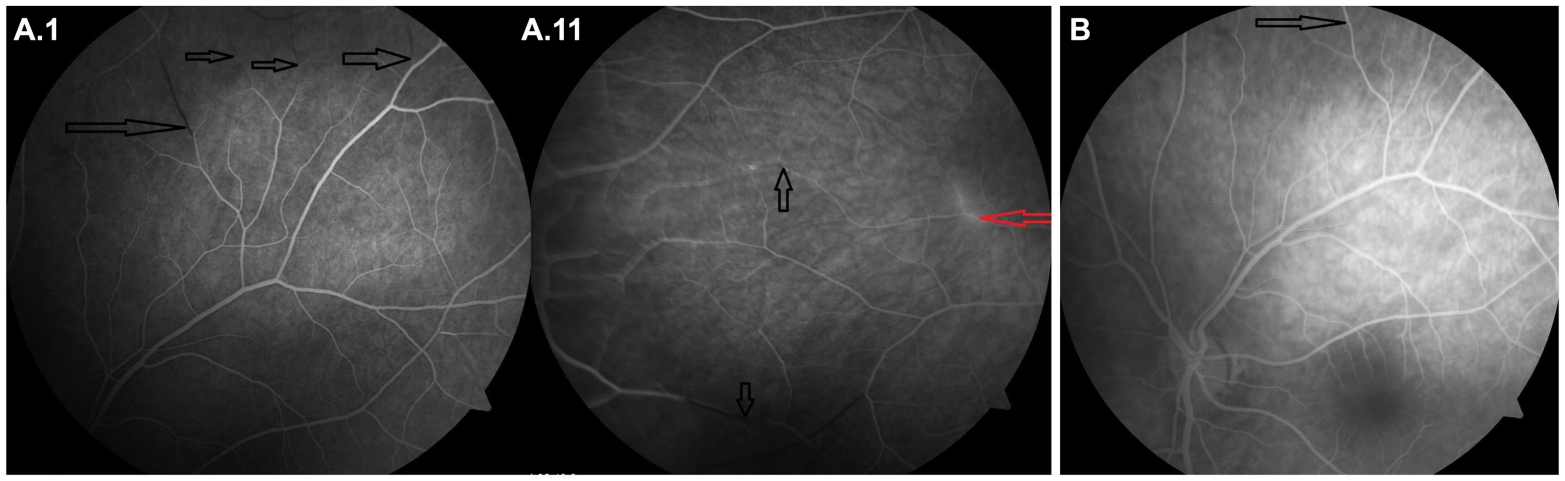
Imaging findings in the case report 2 – Fluorescein angiogram of the left **(A.I)** and right eye **(A.II)** 1.5 months after symptom onset, where the areas of hyperfluorescence mark leakage from inflamed retinal arterioles (red arrow) and the areas of fluorescence decrease correspond to arteriolar occlusion (precapillary plaque) (black arrows). Follow-up fluorescein angiogram (left eye) after 6 months **(B)** showing revascularization of small vessel occlusions present in the previous examination (black arrow); no areas of hyperfluorescence were detected.

The patient was slowly started on oral immunosuppressive medication with a very slow prednisone taper. However, there was a very early relapse with worsening of the clinical condition—right-sided hemiparesis, ataxia and worsening of encephalopathy and speech. Brain MRI scan showed multiple new dot-like areas of diffusion restriction (Figures 3A,B), vessel wall, and leptomeningeal enhancement. The condition was also complicated by active clostridial enterocolitis at the time of the relapse, so she was started on IVIG dosed at 2 g/kg followed by regular administration of IVIG dosed at 1 g/kg every 2 weeks until clinical stabilization. Simultaneously, we stopped oral cyclophosphamide (after 2 weeks in total) and started treatment with rituximab (initial dose of 1 g administered in a 14 days period). After 2 months, when clinical deficit regressed, the interval of IVIG administration was slowly extended to 1.5 g/kg over 3 days every 3 weeks. 6 months after rituximab initiation, the patient remained in remission, her neurological deficit including cognitive impairment slowly improved. Follow-up brain MRI scan detected no new areas of restriction of diffusion, partial regression of the periventricular FLAIR hyperintensities and no enhancement of vessel wall (Figure 3C) or leptomeningeal enhancement in black blood sequences (Figure 2C.II), confirming the remission of the disease. FAG performed after 6 months showed revascularization of small vessel occlusions present in the previous examination; no areas of hyperfluorescence were detected (Figure 4B).

## Review

### Epidemiology and clinical presentation

Susac syndrome (SuS) is a rare disorder, there have been about 450 cases reported worldwide (12). However, its exact prevalence is unknown, and it is thought to be underdiagnosed. A recently published Austrian national cohort reported annual incidence and prevalence of 0.024 and 0.148 per 100,000, respectively (13), while another study suggested annual incidence of up to 0.13 per 100,000 (14). It was first described by John O. Susac in 1979 as an unusual microangiopathy affecting the brain and retina in two women (15). Most published cases originate from North America and Europe. Earlier publications also used the terms “small infarction of cochlear, retinal and encephalic tissue (SICRET) syndrome” (16) and “microangiopathy with retinopathy, encephalopathy and deafness (RED-M)” (17). The disease typically affects younger patients aged between 20 and 40 years with the mean age of onset of 32 and a female to male ratio is 3–3.5:1 (1, 18).

### Symptoms and clinical findings

The classic clinical triad of symptoms involves encephalopathy, visual loss due to branch retinal artery occlusions (BRAOs) and sensorineural hearing loss (1, 19, 20). In the largest cohort study by Dörr et al. (1), the complete triad was present at onset in only about 13% of patients. Nonetheless, it was documented in 85% of cases during the course of the disease with a mean observation period of 41 months.

Central nervous system (CNS) symptoms are the most common clinical manifestation, observed in 67–98% of patients (1, 19). These consist of (12):

- cognitive impairment such as memory deficit, loss of concentration, and executive functions;
- encephalopathy involving behavioral changes, confusion, delusions, apathy and fluctuating or reduced vigilance, up to comatose state;
- focal neurological symptoms such as ataxia, aphasia, motor, or sensory deficits; and
- headache, usually migraine-like and without previous long-term history (within the last 6 months).

About 50% (1) of patients present with visual disturbances comprising of painless partial visual loss with central, paracentral or altitudinal scotoma; further ophthalmic manifestations include photopsias and decreased visual acuity (21, 22). Whereas the typical BRAOs in fluorescein angiography are consistently reported in 95–99% of patients diagnosed with SuS (1, 2, 23), a significant portion of patients may be asymptomatic—in one study, only one third of patients with BRAOs reported visual impairment and about 60% were found to have a visual field defect on examination (19).

The hearing impairment may be unilateral or bilateral, typically affecting low to mid-tone frequencies and may progress to a total hearing loss. Often, hearing disturbance is accompanied by tinnitus and/or vertigo (12).

Other reported symptoms include gait disturbance, nausea and vomiting, oculomotor dysfunction, diplopia, urinary dysfunction, and systemic involvement such as myalgia and arthralgia (1). Spinal involvement is very rare with only few reported cases (24). Until 2022, there were six cases in total of dermatological manifestations in the form of livedo reticularis or livedo racemosa described in literature (7).

In 2015, River et al. (6) reported one case of a possible cardiac involvement in a patient with bradycardia and inverted P waves in II, III and AVF leads which lasted for about 2 weeks. The authors discuss the possible involvement of the conduction system of the heart similarly to other endotheliopathies such as dermatomyositis or brainstem involvement, although a corresponding MRI lesion was not found. This paper presents a second case of bradycardia in Susac syndrome, thus supporting the case for cardiac involvement as part of a broader clinical spectrum.

### Imaging

MRI is the gold standard in evaluating a patient with suspected SuS. White matter lesions of the corpus callosum, deep gray matter lesions and leptomeningeal enhancement form a so-called neuroimaging triad (25). T2-weighted (T2W) or fluid-attenuated inversion recovery (FLAIR) white matter hyperintensities representing microinfarctions of the corpus callosum are characteristic, required for diagnosis according to current criteria (19) and often thought to be pathognomonic (20, 25, 26). However, their prevalence in larger cohorts was only about 78% (1, 3, 27). Microinfarctions develop particularly in the central fibers of the corpus callosum with sparing of the peripheral fibers, thus forming small round lesions referred to as “snowballs” (Figures 2, 3). On the other hand, “spokes” (vertical, radial lesion) and “icicles” (triangular lesions) can be found in the trunk of the corpus callosum (12). In a later course of the disease, these hyperintensities tend to evolve into T1-weighted (T1W) hypointense “holes” (12). Microinfarcts in the internal capsule reminiscent of a “string of pearls” and can be best appreciated in diffusion-weighted imaging (DWI). In general, diffusion restriction signifying acute infarction in various vascular territories can be found in about 50% of cases (3). Traditionally, leptomeningeal enhancement was reported in about 23–44% of patients (1, 3, 25), while some newer data utilizing contrast-enhanced FLAIR sequence rather than the more common contrast-enhanced T1W scans point to a prevalence of up to 56–100% (28, 29). Furthermore, there are descriptions of periventricular lesions, lesions in the basal ganglia, cerebellum, and cerebellar peduncles or brainstem (1).

As for differential diagnosis, callosal lesions can also be found in other disorders, most importantly multiple sclerosis. There, the lesions are typically ovoid, peripheral callosal and callososeptal, perpendicular to the walls of the lateral ventricles or callosal junction and often formed along a central venule, indicating perivascular inflammation (21, 30). Ischemic lesions of corpus callosum are rare but can rarely occur in the splenium. Large, poorly defined, enhancing lesions spreading in a bihemispheric pattern indicate lymphoma or glioblastoma (31). Symmetrical lesions of the splenium and bilateral posterior deep white matter can be seen in hypoxic–ischemic encephalopathy (31) while CLOCC—cytotoxic lesions of the corpus callosum—can be seen in seizures or after sudden cessation of antiepileptic medication. They are visualized as well-circumscribed, small, oval lesions in the midline of the splenium (32). Furthermore, several toxic and metabolic insults may lead to more extensive lesions extending throughout the splenium into hemispheres (31). Callosal lesions, on the other hand, represent a reliable differentiating feature in the differential diagnosis of primary angiitis of the CNS and SuS (33).

Recent findings showed possible utility of black blood contrast-enhanced high-resolution T1W sequences in diagnostics, where it can demonstrate inflammatory changes of the vessel wall due to endothelial injury. Black blood MRI (34) is a non-invasive vessel wall imaging technique able to assess morphology of the vessel wall thickening as well as pattern of enhancement. Specific black blood MRI findings associated with vasculitis and vasculopathy include circumferential and concentric wall thickening and enhancement as well as periarterial and periadventitial enhancement. In a case published by Baskin et al. (35), black blood MRI revealed perivascular contrast enhancement along the T2W hyperintensities in the splenium as well as in the cerebellum. Similarly, our two published cases both feature gadolinium enhancement of vessel walls resulting in a dot-like infarction pattern. Recently, Lotti et al. (36) showed the usefulness of 3D intracranial vessel wall MRI in monitoring the disease activity where it was possibly superior to other techniques in identifying insufficient disease suppression.

### Ophthalmologic examination

Branch retinal arterial occlusions (BRAOs) causing retinal branch ischemia are a hallmark of the disease found in almost all patients and can be identified using FAG. They should be actively searched for in all patients with suspected SuS, even in the absence of clinically manifest visual symptoms and normal fundus examination (18). Even more specifically, the finding of arterial wall hyperfluorescence especially in normal-appearing retinal arterioles in the absence of BRAOs is pathognomonic of the disorder (20). Gass plaques, or retinal arterial wall plaques seen on fundoscopy are another typical finding in SuS, though they can appear in numerous other conditions as well (20). Spectral domain optical coherence tomography (SD-OCT) can demonstrate focal retinal thickening as a correlate to edema in retinal branch ischemia (37).

### Audiogram

Tonal audiogram demonstrates sensorineural hearing loss in low to middle frequencies on one or both ears, which is caused by the infarction of the apical cochlea. While about 37% of patients present with hearing loss at onset, up to 96% tend to develop hearing impairment during the course of the disease (1). Audiogram is eventually pathological in almost all patients who undergo the examination (1, 27).

### Ancillary testing

#### Cerebrospinal fluid

Cerebrospinal fluid testing is often performed as part of the initial workup of patients presenting with acute encephalopathy with focal neurological symptoms. It usually demonstrates mild to moderate protein elevation, mild pleocytosis with predominance of lymphocytes can be found in up to 45% (1). The largest study from the year 2013 reported a mean cell count of 14 (IQR 1–86) (1). CSF-specific oligoclonal band and intrathecal IgG synthesis are rare (1).

#### Vestibular examination

About one fourth of the patients can present with vertigo or balance problems (1). Current diagnostic criteria according to the European Susac Consortium (EuSaC) require a clinical diagnosis of peripheral vertigo supported by a specific vestibular examination such as caloric testing, vestibular evoked myogenic potentials, or videonystagmography (19). However, most currently published cohorts did not report results of vestibular testing. Therefore the exact prevalence of involvement of the vestibular system is not known.

### Diagnostic criteria

In 2016, Kleffner et al. (19) on behalf of EuSac proposed diagnostic criteria for SuS, which were partly validated on 192 published cases. Half of those subjects met all three criteria for a “definite SuS,” further 35% patients would be considered “probable SuS.” Diagnostic criteria proposed by EuSaC are summarized in Table 1.

**Table 1:** Diagnostic criteria of Susac syndrome as proposed by EuSaC in 2016.

	i. Symptoms and clinical findings	ii. Imaging or examination
1. Brain involvement	New cognitive impairment and/or behavioral changes and/or new focal neurological symptoms and/or new headache	Typical findings in cranial MRI–hyperintense, multifocal, round small lesions, at least one of them in the corpus callosum (“snowball”) in T2W (or FLAIR) sequences.
(at least one symptom and one MRI finding required)		
2. Retinal involvement	Not required	BRAOs or AWH in fluorescein angiography or characteristic signs of retinal branch ischemia in fundoscopy or SD-OCT
(only ophthalmological finding required)		
3. Vestibulocochlear involvement	New tinnitus and/or hearing loss and/or peripheral vertigo	Hearing loss supported by an audiogram; vestibular vertigo supported by specific diagnostics
(clinical finding supported by corresponding examination required in case of hearing loss and vertigo)		

Definite diagnosis: all three criteria met. Probable diagnosis: two out of three criteria met. Possible diagnosis: only one criterion met. AWH, Arterial wall hyperfluorescence; BRAOs, Branch retinal artery occlusions; FLAIR, Fluid-attenuated inversion recovery; T2W, T2-weighted; and SD-OCT, Spectral domain optical coherence tomography.

### Treatment

The treatment of SuS remains very challenging for several reasons. Mainly because of the heterogeneity of the clinical presentation, variable disease course and severity, and the rarity of the disease. Randomized clinical trials or prospective studies do not exist, so there is no evidence-based treatment (9, 12, 38, 39). Treatment strategies are based on similarity with another autoimmune microvascular endotheliopathy with microinfarctions affecting skin, muscles, and gastrointestinal tract—the juvenile dermatomyositis (9, 12). Because of the missing prospective and randomized clinical trials, the guidelines created by experts are based on reviews of case series and reports, clinical experience of the guideline authors, follow-up of patient cohorts and known pathogenesis of the disease (1, 9, 12).

The recommendations focus mainly on the initiation of the treatment and first year maintenance strategy. They include several immunomodulatory and immunosuppressive medications with an emphasis on corticosteroids and IVIG. They present several aggressive treatment protocols and algorithms with combinations of immunosuppressive and immunomodulatory agents depending on the primary affected organ and disease severity (9, 12, 20, 38, 40).

Generally, the treatment initiation in active moderate to severe SuS with CNS-predominance includes a high dose of intravenous corticosteroids (usually 3–7 g of methylprednisolone) followed by oral prednisone (1 mg/kg/day) with very slow tapering. Clinical practice shows that the disease is very responsive to corticosteroids, although with frequent relapses (9, 12). Therefore, the treatment protocols include IVIG (2 g/kg administered during a 2-day period) with regular infusions every 2 weeks. IVIG is an additional protection and similarly to a very slow prednisone tapering, IVIG administration intervals should be extended very carefully to prevent relapse (9). IVIG treatment should last about 12 months.

As an alternative to IVIG, plasmapheresis was proposed, albeit with little clinical experience (12, 39). Successful use of subcutaneous immunoglobulin instead of regular intravenous infusions has also been described (41). In patients with severe and extremely severe CNS-predominant SuS, early addition of rituximab (as an initiation 2 × 1 g with a gap of 14 days, then repeated after 4–6 and 12 months) is strongly recommended (9, 12, 42). The clinical experience confirms that it is efficient, although only few cases were reported (8, 14, 27, 42–46). Equally, ocrelizumab as an alternative may be considered despite the lack of clinical experience (9). There are only several case reports of other monoclonal antibodies, such as infliximab (47) or natalizumab (12). The number of patients managed with natalizumab is very low, and relapse after administration has been described (48). Aggressive immunosuppressants such as intravenous cyclophosphamide are reserved for patients with extremely severe disease course due to the side effect profile (9, 12). The recommended therapeutic regimen is based on a minimum of two intravenous pulses of 10 mg/kg, 2 weeks apart, continued until clinical stabilization (9). Alternatively, mycophenolate mofetil may be used (9, 12, 38).

After stabilization, chronic maintenance treatment is recommended. The long-term treatment options are even more ambiguous than active disease management. Several immunosuppressive medications can be chosen, depending on patient characteristics and the clinic’s experience. Most current guidelines recommend mycophenolate mofetil or mycophenolate mofetil and tacrolimus with addition of Rituximab in the most severe cases while slowly tapering corticosteroids and IVIG, although data regarding comparative efficacy are missing (9).

In mild to moderate SuS course affecting CNS, a combination of prednisone, IVIG every 3–4 weeks and mycophenolate mofetil is usually sufficient. In case of more severe symptoms, experts advise to consider adding rituximab into the mix (9).

In retina- or ear-predominant disease, the management approach usually resembles the treatment of mild to moderate CNS SuS according to the disease severity. In serious cases of retinal vasculopathy, rituximab or cyclophosphamide should be strongly considered (9). In disease affecting predominantly inner ear, local trans-tympanic dexamethasone injection is a suitable option (49).

### Prognosis

The long-term prognosis of patients with SuS depends on the variability of clinical presentation, disease course, its severity and response to treatment. Clinical course of the disease may be monophasic, polycyclic (or relapsing–remitting), or chronic (18, 50). In most cases, the disease is self-limiting with an average disease duration of about 2 years (9, 12), although relapses after decades of remission have been reported (51, 52). Due to many unpredictable variables, anticipating the prognosis is impossible and early aggressive treatment is the only way to prevent long-term disability (9).

## Discussion

This paper describes two vastly different cases representing broad clinical variability of the disease. In patient 1, a milder manifestation of the disease was observed, consisting of encephalopathy and vertigo, with subsequent paraclinical investigations leading to the diagnosis of definite SuS. This patient improved after one pulse therapy of methylprednisolone and remained stable on azathioprine.

Patient 2 experienced a very different clinical course. After initial episodes of recurrent left-sided sensorimotor deficit, she developed severe encephalopathy and lasting left-sided hemiparesis. The subsequent investigations again confirmed the diagnosis of SuS; however, the treatment was much more challenging in contrast to patient 2. Two subsequent pulses of methylprednisolone did not achieve remission and plasmapheresis was employed.

Plasmapheresis is used less commonly in SuS. Limited evidence available in literature points to reasonable effectiveness of this treatment modality (27, 44). In this patient, it led to stabilization, even though only four cycles were performed due to the patient developing asymptomatic coagulopathy. She was then started on cyclophosphamide and remained stable for several weeks, after which she relapsed. This time, due to an active clostridial enterocolitis, she was successfully treated with IVIG and rituximab.

Interestingly, this patient also presented with recurrent asymptomatic bradycardia which resolved after the aforementioned immunosuppressive regimen. According to our knowledge this is only second reported case. As previously published by River et al. (6), the bradycardia may be a consequence of the conduction system of the heart similarly to other endotheliopathies such as dermatomyositis or brainstem involvement. The cardiac involvement only supports the variability of manifestations.

In both presented cases, black blood MRI sequence was utilized and revealed gadolinium enhancement of small intracranial vessel walls corresponding with point infarctions of the brain parenchyma along with leptomeningeal enhancement. This highlights the utility of this diagnostic method which could become a standard part of the workup in suspected SuS.

## Conclusion

Susac syndrome is a rare and complex neurological disorder primarily affecting small blood vessels in the brain, retina, and inner ear. Diagnosis can be very challenging due to its rarity and broad clinical variability. We highlight the benefit of black blood MRI of the vessel wall that can be very useful diagnostic tool and provides us also important information according to disease activity and treatment efficacy. Despite its rarity, the awareness of Susac syndrome may be of uttermost importance since it ultimately is a curable condition. If diagnosed in a timely manner, early intervention can substantially improve the outcomes of our patients.

## Data availability statement

The raw data supporting the conclusions of this article will be made available by the authors, without undue reservation.

## Ethics statement

Ethical approval was not required for the study involving humans in accordance with the local legislation and institutional requirements. Written informed consent to participate in this study was not required from the participants or the participants’ legal guardians/next of kin in accordance with the national legislation and the institutional requirements. Written informed consent was obtained from the individual(s) for the publication of any potentially identifiable images or data included in this article.

## Author contributions

MC: Conceptualization, Resources, Writing – original draft, Writing – review & editing. JŠ: Conceptualization, Writing – original draft, Writing – review & editing. VV: Writing – original draft. MD: Resources. ID: Resources. JV: Writing – review & editing. RH: Writing – review & editing. MZ: Resources, Writing – review & editing. VČ: Resources, Visualization, Writing – review & editing. JB: Writing – review & editing. VB – Writing - review & editing. DK: Writing – review & editing. PA: Writing – review & editing. PF: Writing – review & editing. VW: Conceptualization, Project administration, Supervision, Writing – review & editing.
